# Supplementary material for: Evaluation of Robotic Systems on Cytotoxic Drug Preparation: A Systematic Review and Meta-Analysis
Source: Medicina (Kaunas). 2023 Feb 22;59(3):431. doi: 10.3390/medicina59030431 (PMC10056266; doi:10.3390/medicina59030431)
Supplement: Supplementary file 1 [file medicina-59-00431-s001.zip › Table S1.pdf]

Table S1. – Search strategy

PubMed	
#1	Search: compounding<br>"compounded"[All Fields] OR "compounder"[All Fields] OR "compounders"[All Fields] OR "compounding"[All Fields] OR "compoundings"[All Fields]
#2	Search: (robotics) OR (automation)<br>((((((("robot"[All Fields] OR "robot s"[All Fields]) OR "robotically"[All Fields]) OR "robotics"[MeSH Terms]) OR "robotics"[All Fields]) OR "robotic"[All Fields]) OR "robotization"[All Fields]) OR "robotized"[All Fields]) OR "robots"[All Fields]) OR (((((("automate"[All Fields] OR "automated"[All Fields]) OR "automates"[All Fields]) OR "automating"[All Fields]) OR "automation"[MeSH Terms]) OR "automation"[All Fields]) OR "automations"[All Fields])
#3	#1 AND #2 / n=369
EMBASE	
#1	Compounding
#2	Robotics or automation
#3	#1 AND #2 / n=143
Cochrane	
#1	(compounding) AND (robotor automation) / n=415
