# Supplementary material for: Evaluation of Robotic Systems on Cytotoxic Drug Preparation: A Systematic Review and Meta-Analysis
Source: Medicina (Kaunas). 2023 Feb 22;59(3):431. doi: 10.3390/medicina59030431 (PMC10056266; doi:10.3390/medicina59030431)
Supplement: Supplementary file 1 [file medicina-59-00431-s001.zip › Table S2.pdf]

Table S2. Secondary outcomes of systematic review

Study	Robot brand	Study design	Outcomes	Results
Bhakata et al, USA, 2018 [18]	i.v.STATION	Quasi-experimental study	Cost	Robot saves $129,477 per year for preparing 20 doses per day
Seger et al, USA, 2012 [2]	CytoCare	Quasi-experimental study	Cost	Robot saves $115,500 ancillary cost while preparing 16,500 antineoplastic agents
			Failure	45 mechanical failures out of 110 preparations
			Number of safety events	82 out of 1421 preparations in manual vs 35 out of 972 preparations in robot
Heloury et al, France, 2019 [22]	KIRO	Quasi-experimental study	FMECA index†	1060 in manual vs 656 in robot
Chen et al, Taiwan, 2013 [19]	CytoCare	Case-series study	FTE‡ saving	No noticeable difference
Yaniv et al, USA, 2013 [16]	APOTECAchemo	Case-series study	Number of errors§	Mechanical error: 155<br>Human error: 12<br>Interface/IT error§§: 4

† FMECA (failure mode, effects and criticality analysis), ‡ FTE (full-time equivalent), § Total 7384 patient doses §§Error caused by robotic system
