# Supplementary figures and images for: Evaluation of Robotic Systems on Cytotoxic Drug Preparation: A Systematic Review and Meta-Analysis
Source: Medicina (Kaunas). 2023 Feb 22;59(3):431. doi: 10.3390/medicina59030431 (PMC10056266; doi:10.3390/medicina59030431)

## Slide 1
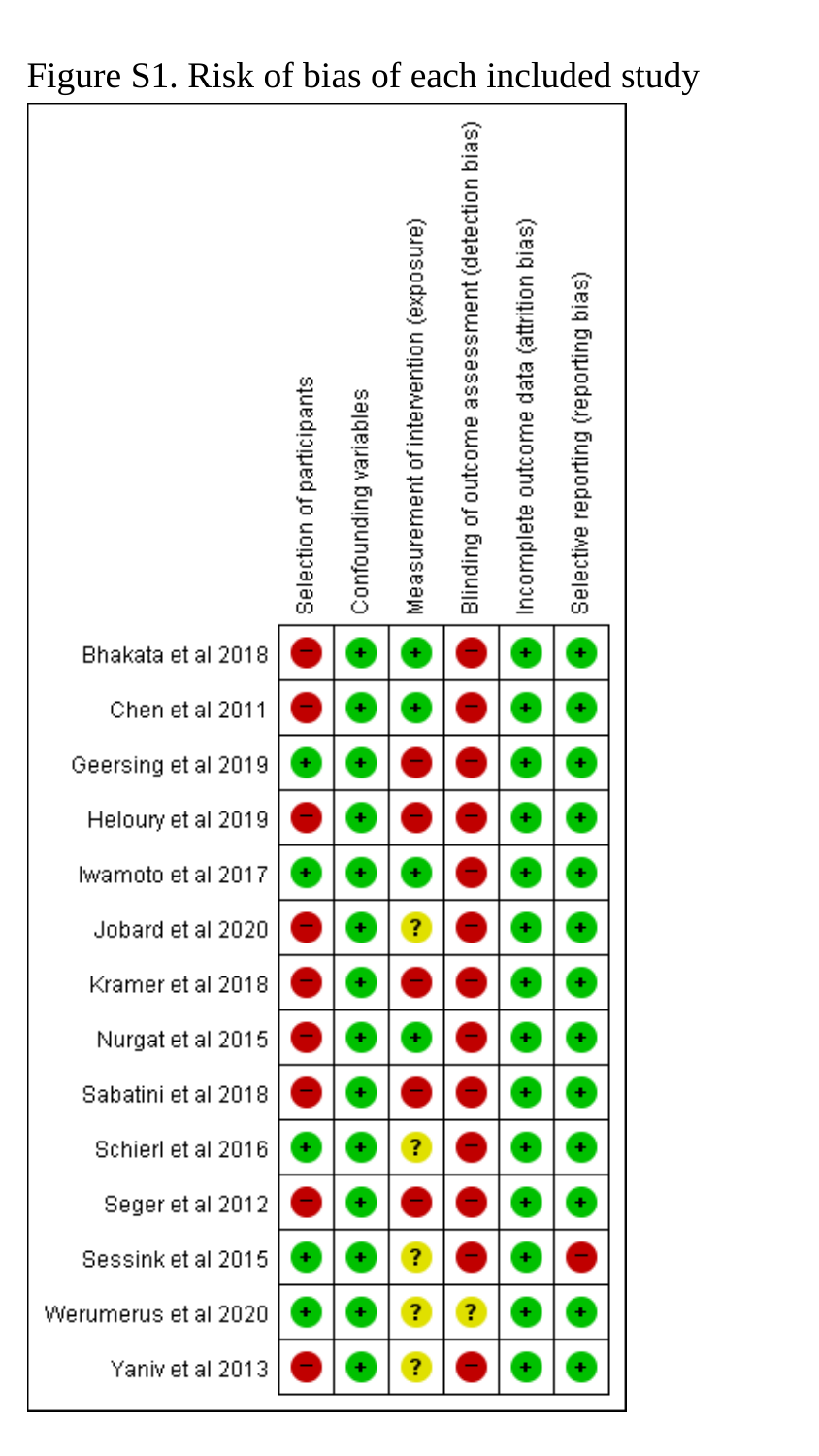

Figure S1. Risk of bias of each included study

Supplement: Supplementary file 1 [file medicina-59-00431-s001.zip › Figure S1.pptx]
